# High Versus Low-Energy Extracorporeal Shockwave Therapy for Chronic Lateral Epicondylitis: A Retrospective Study

**DOI:** 10.3390/jfmk9030173

**Published:** 2024-09-22

**Authors:** Gabriele Santilli, Francesco Ioppolo, Massimiliano Mangone, Francesco Agostini, Andrea Bernetti, Sara Forleo, Sara Cazzolla, Anna Camilla Mannino, Alessio Fricano, Antonio Franchitto, Samanta Taurone, Antonello Ciccarelli, Marco Paoloni

**Affiliations:** 1 Department of Movement, Human and Health Sciences, Division of Health Sciences, University of Rome “Foro Italico”, 00135 Rome, Italy; 2 Department of Anatomical and Histological Sciences, Legal Medicine and Orthopedics, Sapienza University, 00185 Rome, Italy; 3 Department of Biological and Environmental Science and Technologies, University of Salento, 73100 Lecce, Italy

**Keywords:** extracorporeal shockwave therapy ESWT, lateral epicondylitis, lateral elbow tendinopathy, upper limb, rehabilitation, minimally invasive, physiotherapy, treatment success rate, visual analog scale, energy level, pain, upper limb, conservative treatment, PRTEE

## Abstract

**Background**: Chronic lateral epicondylitis (LE), also known as tennis elbow, affects 1–3% of the population, primarily those over 40 years old. Most cases resolve with conservative treatments, but some require more advanced interventions. Extracorporeal shockwave therapy (ESWT) has emerged as a non-surgical treatment option, utilizing either low- or high-energy levels to alleviate pain and improve function. **Objective**: This study aimed to compare the efficacy of low-energy versus high-energy ESWT in the treatment of chronic LE, focusing on pain relief and functional improvement. **Methods**: A retrospective observational study was conducted including patients treated for chronic LE between 2021 and 2024. Participants were divided into two groups: low-energy ESWT (0.10 mJ/mm^2^) and high-energy ESWT (0.20 mJ/mm^2^). Both groups received 2400 pulses at a frequency of 6 Hz once a week for three weeks. Pain and functional outcomes were measured using a visual analog scale (VAS) and the Patient-Rated Tennis Elbow Evaluation Questionnaire (PRTEE) at the baseline, three months (T1), and six months (T2) post-treatment. **Results**: Forty-six patients participated, with 24 in the low-energy group and 22 in the high-energy group. Baseline demographics and clinical characteristics were similar across groups. At T1 and T2, the low-energy group showed significantly greater reductions in the VAS scores (T1: 4.45 ± 0.8 vs. 3.6 ± 1.7, *p* = 0.04; T2: 3.2 ± 1.2 vs. 2.1 ± 1.1, *p* = 0.004) and PRTEE scores (T1: 34.3 ± 6.9 vs. 26.8 ± 11.9, *p* = 0.03; T2: 25.3 ± 6 vs. 17.6 ± 9, *p* = 0.005). Significant treatment–time interactions were observed for both the VAS and PRTEE scores, indicating sustained improvements in the low-energy group. **Conclusions**: Low-energy ESWT was more effective than high-energy ESWT in treating chronic LE, providing greater and longer-lasting pain relief and functional improvement. These findings suggest that low-energy ESWT should be preferred in clinical practice for managing this condition. Future research should focus on larger sample sizes and randomized controlled trials to confirm these results and explore the underlying mechanisms of differential efficacy between energy levels.

## 1. Introduction

Tennis elbow or lateral epicondylitis (LE), also referred to as proximal wrist extensor tendinopathy, manifests as pain either directly at or slightly below the lateral humeral epicondyle within the tendon of the proximal wrist extensor. It affects around 1–3% of the population, primarily those over 40, with equal prevalence between genders. Most cases (70–90%) experience spontaneous remission or respond well to conservative treatment within a year [1], involving rest, nonsteroidal anti-inflammatory drugs (NSAIDS), orthosis, physical therapy, and corticosteroid injections [2]. The normal anatomical architecture of the tissues around the lateral epicondyle is complex and continues to be a focus of study [3]. Based on macroscopic and microscopic observations in 40 fresh-frozen cadavers, the tendons of the extensor carpi radialis brevis (ECRB) and extensor digitorum communis (EDC) were found to be indistinguishable at the osteotendinous origins [4]. The thin, tendinous EDC and ECRB insertions are particularly vulnerable to injury [5]. Ultrasonographic assessment at the humeral epicondyles enables accurate EDC and ECRB insertional identification and differentiation [6]. Lateral elbow tendinosis is part of the larger family of tendinosis conditions. Several review articles have commented on the term “tennis elbow” because the prevalence of LE in tennis players contributes to only 5% of all LE cases [7]. The LE etiology has long been associated with performing forceful, highly repetitive physical work tasks, particularly with prolonged non-neutral hand and wrist postures [8]. High HbA1c and blood glucose levels have also been associated with LE [9], as has female sex [10]. Smoking cessation significantly reduces LE risk [11], and hypercholesterolemia incidence is higher in LE patients than in healthy controls [9]. 

The diagnosis of LE is mainly clinical [10]. The precise evaluation of the patient’s history and a complete examination are mandatory. The key finding of the physical examination is local tenderness over the origin of the ECRB at the lateral epicondyle, and symptoms are often reproduced through provocative tests [1]. The most widely accepted and used tests are Cozen’s, Mill’s, and Maudsley’s [11], and there was recently a new test called “the selfie test”, where the patient is instructed to hold a cell phone with their elbow fully extended and flex their wrist, independently pressing their thumb on the phone screen or top button. A positive test is indicated by the presence of pain in the lateral aspect of the elbow joint, which could be a valuable addition to the diagnostic process [10]. As the diagnosis is clinically based, imaging is often unnecessary, although it can assist in more complex cases [12]. Ultrasonography (US) has been proven to have high sensitivity but low specificity in the diagnosis of LE, and is considered the most useful tool; however, it should not be used in isolation, but rather as a complementary component of the overall assessment [12,13,14].

Recent studies have highlighted the effectiveness of corticosteroid, platelet-rich plasma, autologous blood products, or botulinum toxin injections in relieving pain and enhancing functionality for patients unresponsive to medication and seeking to avoid surgery [2]. Surgery, whether arthroscopic or open, is typically recommended for patients with LE resistant to conservative treatments due to excessive tension, repetitive micro-trauma, and degenerative changes in the extensor carpi radialis brevis (ECRB) tendon [15]. Extracorporeal shockwave therapy (ESWT) is gaining popularity as a non-surgical option for treating various musculoskeletal conditions including LE. It aims to alleviate pain and improve function by stimulating tissue healing and activating nerve fibers [16]. Through the stimulation of vascular endothelial growth factor and nitric oxide synthase [17,18], ESWT can improve the blood supply to, and therefore tissue regeneration at the tendon–bone junction. The resolution of injured tendon edema, swelling, and inflammatory cell infiltration has also been reported with ESWT use [19]. The use of ESWT also increases the expression of TGFβ1 and IGF-1, triggering the release of lubricin, which stimulates tenocyte growth, proliferation, and facilitates tendon repair, gliding, and tissue healing [20]. Recent meta-analyses and systematic reviews suggest that ESWT is a safer alternative compared to other techniques and is increasingly recommended as a noninvasive option [21]. Additionally, studies indicate that ESWT yields better long-term outcomes in terms of grip strength and pain relief compared to corticosteroid injections, as demonstrated in recent level 1 meta-analyses [22].

ESWT can be separated into low-energy flux density (<0.12 mJ/mm) and high-energy energy flux density (≥0.12 cmm) treatments regardless of the types of shockwave generators, and these energy levels have been widely adopted to treat different tendinopathies such as supraspinatus tendinopathy [23] or fasciopathies such as plantar fasciitis [24] or other diseases such as chronic heel pain or myofascial pain syndrome [25,26,27].

Biological tissues interact with chemical substances (pharmaceuticals, in particular), but also with physical energy [28]. In the case of pharmaceuticals, those used in modern medicine are becoming ever more precise in aiming at specific types of molecules [29], which is what makes it possible to cure specific illnesses. For every pharmaceutical, the dosage is generally well-defined in terms of quantity and temporal duration for the cure (the interval between one administration and the next and the total number of days of administration). The same cannot be said for instrumental physical therapies, which, often for the same illness, propose protocols that are quite different from one another in terms of the number of sessions, the intervals between one session and the next, and the intensity of the physical energy administered. For some electro-medical devices, moreover, even though the intensity of the physical energy administered remains the same, the way the device interacts with the patient will result in a greater or lesser release of energy into the tissues, for example, regarding the width of the contact surface between the skin and handpiece. This is the case with sound waves. High pressure mechanical waves propagate directly when the tissue density is uniform, however, the ESWT probe angle influences how much energy is released into the region of interest

To date, it is not yet clear which energy level, or which energy or power level, is the most effective for pain relief and clinical improvement of elbow function in LE using ESWT. Only one study has compared the two energy levels in LE, but since NSAIDs were used during the therapy in both groups [30], it cannot be considered as an accurate assessment of the effect of ESWT alone for LE [31]. Research protocols vary in recommendations for the adjuvant treatment with physical therapy, eccentric loading, stretching, and the use of NSAIDs [31]. Therefore, we conducted an observational study to compare the healing effects of high-energy ESWT alone with low-energy ESWT alone in the therapy of chronic lateral epicondylitis. The aim of the study was to determine whether low-energy or high-energy ESWT is more effective for reducing elbow pain and improving function in patients with LE.

## 2. Materials and Methods

### 2.1. Study Design and Population

**Figure 1:**
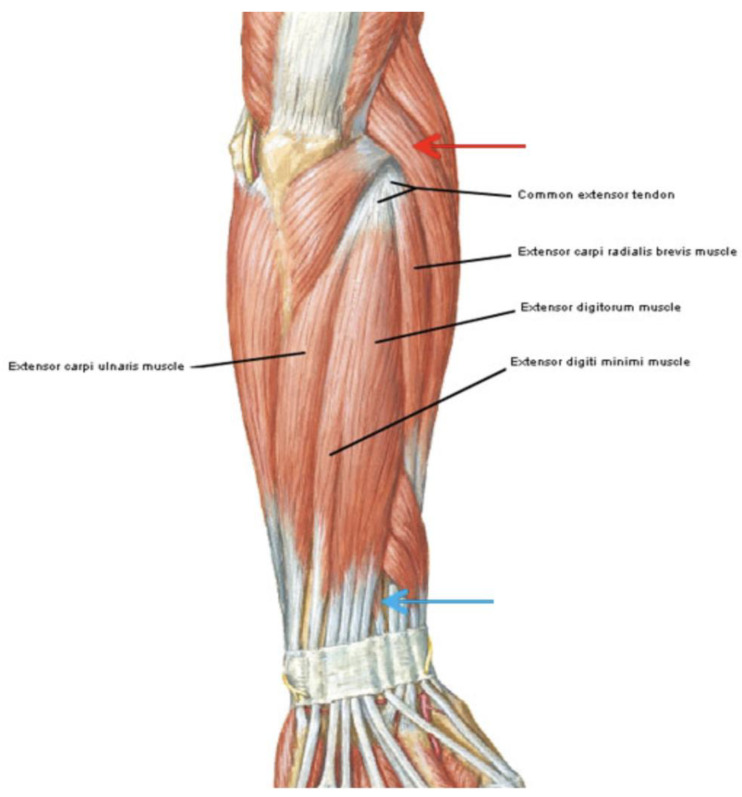
Local pain in the area of the lateral epicondyle (red arrow) and distal course of the extensor tendons (blue arrow).

**Figure 2:**
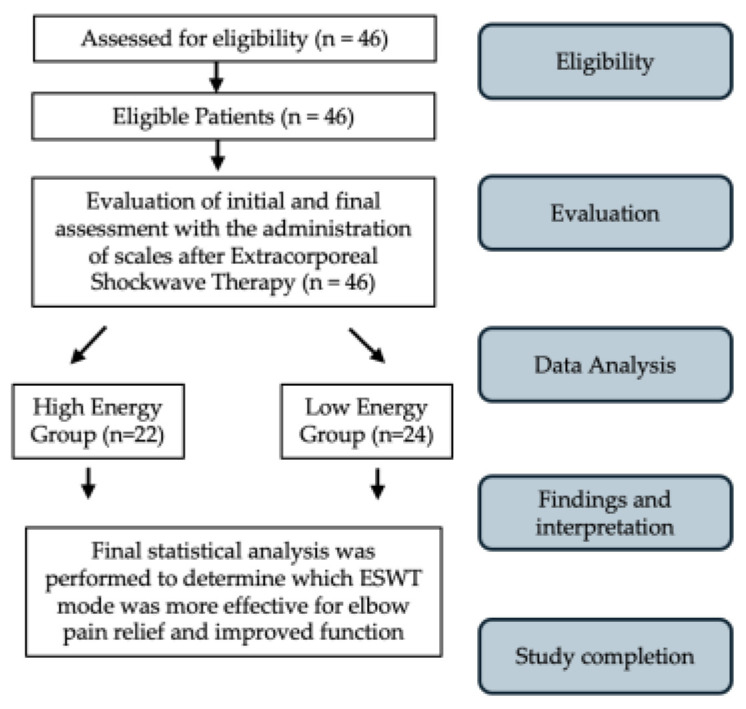
Flow diagram of the study.

This observational study followed good clinical practice and the ethics of the Helsinki Declaration, approved by La Sapienza University’s Institutional Review Board (Prot. 0000/2024—Approval Date: 15 February 2024). Informed consent forms were signed by all patients, and the data were anonymized. All participants provided signed informed consent before the study, which included a specific section regarding the processing of their personal data for research purposes, ensuring anonymization to safeguard their privacy. The data of patients treated for symptomatic lateral elbow pain between 2021 and 2024 were retrospectively collected and analyzed. LE was diagnosed based on clinical symptoms, physical examinations, and imaging studies. To be study eligible, patients had to be between 25 and 85 years old, have had pain in the epicondyle are of the lateral epicondyle (Figure 1), with symptoms over the last 3 months, with reduced range of motion (ROM), and present with a positive tendinopathy epicondylitis test. Patients were excluded if they had marked atrophy or weakness at any forearm muscles, if they had previously undergone elbow-region surgery, if they had recent corticosteroid or nerve blockage injections, if there was a tumor in the treatment area, if they were pregnant, or if they had any coagulation abnormalities. All eligible patients completed a demographic and clinical questionnaire that assessed age and gender. The following scales were administered at T0 and at T1 after three months and at T2 after 6 months from ESWT: A visual analog scale (VAS) [32] and Patient-Rated Tennis Elbow Evaluation Questionnaire (PRTEE) [33]. All ESWT applications and survey administration were performed by the primary investigator, a physician specialized in physical medicine and rehabilitation. Participants were instructed not to consume NSAIDs during the study period, and they were asked to report any intake including self-medication on demand. The flow diagram of the study is shown in Figure 2.

### 2.2. Intervention

The study protocol used was in line with the current state-of-the-art for treating LE with ESWT performed by the principal authors using the Modulith SLK system (Storz Medical, Tagerwilen, Switzerland), with an electromagnetic extracorporeal shockwave generator equipped with an in-line ultrasound positioning system on the target zone. The following parameters were followed. Firstly, ultrasonographic localization of the region of interest was performed, and secondly, the focus was positioned according to the site of the subject’s maximum local pain at treatment initiation [34]. All treatments were performed with no local anesthesia. Participants underwent ESWT with the patient’s forearm positioned neutrally and their elbow bent at a 90-degree angle while sitting on a bed [35]. The data of patients who underwent either high-energy or low-energy ESWT were collected from a pre-existing dataset. Specifically, this included the number of patients and therapeutic characteristics as follows: low-energy level of 0.10 mJ/mm^2^ (Low-en-g), with 24 patients, and at a high-energy level of 0.20 mJ/mm^2^, with 22 patients (High-en-g). Both groups received 2400 pulses at a frequency of 6 Hz once a week for three weeks. The VAS and PRTEE were administered before treatment, at T1 after three months, and at T2 after six months from ESWT.

#### Outcomes

All ESWT applications and survey administration were performed by the primary investigator, a physician specialized in physical medicine and rehabilitation. The visual analog scale (VAS) comprised a 100-mm horizontal line, with “no pain” denoted at the left end (score: 0) and “pain as severe as possible” at the right end (score: 10). Patients were instructed to place a hatch mark on the line corresponding to their current pain level, both at rest and during their most painful movement. The VAS score was subsequently determined by measuring the distance in millimeters between the left endpoint and the patient’s mark [32].

The validated Italian version of the PRTEE questionnaire was used in this study. This is a specific assessment tool in LE patients consisting of 15 items and three subgroups (pain, special activities, and daily living activities). Higher scores indicate increased pain and functional disability (0 = no disability) [36,37].

### 2.3. Statistical Analysis

Power analysis was performed using G*Power (v.3.1.9.2, developed by Franz Faul and colleagues at the University of Kiel, Germany). Based on the study by Riaz S et al. [38], a desired statistical power of 90% was assumed to detect a difference of 1.5 points in the VAS-pain score, using a two-tailed *t*-test with Bonferroni corrections. The acceptable precision level was determined with a standard deviation (SD) of 1.5 points. A confidence level of 95% (α = 0.05) was specified, and an effect size of 0.93 was considered to determine the magnitude of practically significant differences. With these parameters, a sample size of 21 participants per group was calculated to be sufficient. Descriptive statistics (mean and standard deviations) were used to describe the characteristics of the two groups as well as the outcome variables before and after intervention using paired samples *t*-tests to provide an initial understanding of the data. Chi-square tests were used to determine the group frequency differences based on gender. The normality of these variables was assessed using the Shapiro–Wilk test for continuous variables. After confirming the normal distribution of the variables in the two groups, a repeated measures ANOVA was conducted to evaluate the change in VAS and PRTEE from the baseline at the two follow-up time points after treatment. We used a mixed two-way ANOVA, with time as the within-subject factor and group as the between-subject factor. In cases where a significant ANOVA result was found (*p* < 0.05), we performed post hoc analysis to determine which specific group differences contributed to the significance. Specifically, we used the Bonferroni corrections for the post hoc test to adjust for multiple comparisons and minimize the risk of type I errors. Additionally, bivariate correlations were performed to assess the relationships between variables, specifically, Pearson’s correlation was applied for continuous variables, while Spearman’s rho correlations were used for ordinal variables. *p* < 0.05 was considered statistically different. A cut-off for correlations was considered as follows: values between 0.3 and 0.7 indicated a moderate positive linear relationship, while values between −0.3 and −0.7 indicated a moderate negative linear relationship [39].

## 3. Results

### 3.1. Patient Demographic and Clinical Characteristics

All patients participated in the 3- and 6-month follow-ups (Table 1). Demographic and clinical measurement characteristics were comparable between groups

Descriptive statistics (mean and standard deviations) were used to describe the outcome variables before and after intervention, using paired samples *t*-tests as shown in Table 2.

Repeated-measures 2-way ANOVAs for VAS scores showed no significant differences at the baseline between the two groups. At T1 and at T2, a significant effect of treatment was observed, with the low-power group exhibiting a lower VAS (T1: 4.45 ± 0.8 vs. 3.6 ± 1.7; *p* value 0.04) (T2: 3.2 ± 1.2 vs. 2.1 ± 1.1; *p* value 0.004), respectively. Additionally, a significant treatment–time interaction was observed (F(2, 88) = 7.56, *p* < 0.001, with a partial eta squared η^2^ = 0.15 indicating a large effect size [40]. A strong, significant change in test performance over time was observed in both groups (*p* value < 0.01). Following the repeated-measures ANOVAs for VAS scores, post hoc tests were conducted using the Bonferroni correction to account for multiple comparisons. The results showed that at T1, the difference between the groups was significant (*p* < 0.05), but at T2, more significant differences were found (*p* = 0.004), with the low-power group reporting significantly lower VAS values compared to the high-power group (mean difference = 1.094; 95% CI: 0.368–1.821). These findings confirm a stronger treatment effect over time, consistent with the significant treatment–time interaction observed in the ANOVA.

Furthermore, we conducted a repeated-measures 2-way ANOVA for PRTEE scores, which showed no significant differences at the baseline between the two groups. At T1 and T2, a significant effect of treatment was observed, with the low-power group exhibiting a lower PRTEE (T1: 34.3 ± 6.9 vs. 26.8 ± 11.9; *p* value 0.03) (T2: 25.3 ± 6 vs. 17.6 ± 9; *p* value 0.005), respectively. A significant treatment–time interaction was observed (F(2, 88) = 7.56, *p* < 0.001, with a partial eta squared η^2^ = 0.07 indicating a moderate effect size [40]. A strong, significant change in test performance over time was observed in both groups (*p* value < 0.01). To further explore these differences, post hoc tests with Bonferroni correction were conducted. The results revealed significant differences between groups at T1 and T2, with mean differences of 7.476 (*p* = 0.03) and 7.718 (*p* = 0.005), respectively, favoring the low-power group. The 95% confidence intervals for these differences were [0.768, 14.184] at T1 and [2.437, 13.000] at T2, indicating a statistically significant improvement in the low-power group compared to the high-power group at these time points. The relationship between variables was evaluated through bivariate correlations using Pearson correlation coefficients. Furthermore, concerning the correlation with the binary variable “group”, Spearman’s rho was performed, the results of which are presented in Table 3.

### 3.2. Safety

Throughout the study period, no significant adverse effects related to ESWT were observed or reported by the participants.

## 4. Discussion

Despite the extensive use of ESWT worldwide, studies on the dose relationship between the intensity and the biological effects of ESWT remain inadequate [27]. The treatment with ESWT for LE has been included in the guidelines of the International Society for Medical Shockwave Treatment (ISMST) [41]. However, recent systematic reviews and meta-analysis on the topic have not fully clarified the role of this therapy in treating this condition. Instead, they show conflicting results in effectiveness and do not agree on which types of shockwaves to use or the treatment protocol [21,42]. In some studies, regarding the treatment of LE with ESWT, high energy was used [43,44], while in others, low energy was used [45,46]. The therapy with ESWT involves administering sound waves with pressure peaks that can reach 35–120 MPa and its effects, even at the molecular level, depend on the energy delivered to the specific area, based on a parameter known as the energy flux density (EFD) [47,48]. Some studies have been developed to investigate the effects of differences in power, for example, on plantar fasciitis [49].

This study aimed to specifically assess the difference between the two EFDs in the treatment of LE with ESWT alone. In our study, two different energy levels, based on past studies [23], were considered: the low-energy group (Low-en-g) 0.10 mJ/mm^2^ and high-energy group (High-en-g) 0.20 mJ/mm^2^, while keeping the number of sessions (three) and administered shocks (2400) constant at 4 Hz. There is no consensus as to the appropriate EFD, number of sessions, and SWT impulses, and it is not known whether and, if so, to what degree a correlation exists between decreased pain and functional recovery, on the one hand, and the resorption of calcific deposits, on the other hand [50,51,52,53,54,55]. In supraspinatus calcific tendinopathy and chronic heel pain conditions, it has been demonstrated that high energy produced greater clinical improvement [17,54,56,57].

In our study, the demographic characteristics of the participants including age and gender distribution were homogenous between the two groups, suggesting that the differences observed in outcomes were attributable to the treatment modalities rather than the confounding variables. The baseline VAS and PRTEE scores also showed no significant differences, reinforcing the comparability of the two groups at the start of the intervention.

The results suggest a greater improvement in pain and functional outcomes with low-energy ESWT. However, these results need to be confirmed in randomized controlled trials.

Improvements observed over time were significantly influenced by the type of ESWT administered. This interaction underscores the efficacy of low-energy ESWT in LE in promoting more pronounced improvements at 3 and at 6 months in terms of VAS score and PRTEE score.

The correlation analyses (Table 3) revealed a strong relationship between the PRTEE scores and patient-reported measures (VAS scores), indicating that pain reduction is closely associated with improvements in functional abilities, however, these results need to be confirmed in randomized controlled trials.

We did not detect statistically significant relationships between patient age or gender and pain or function measurements.

Lacking several specific data on the difference in energy levels of ESWT in LE, it was necessary to refer to more extensively studied rotator cuff tendinopathy data. These studies indicated that high-energy application, compared to low-energy, resulted in greater calcific deposit resorption, as demonstrated in cases of rotator cuff calcific tendonitis [17,54,56]. Conversely, basic research studies have shown that low-energy applications induce numerous biological effects such as the stimulation of angiogenesis [41,57] and an increased synthesis of nitric oxide, which has been demonstrated in vitro to provide anti-inflammatory effects by flushing out inflammatory mediators [58]. Furthermore, another aspect to consider, as cited in the ISMT guidelines [59], regards the power or intensity, which is quantified as the EFD corresponding to the pressure (P) at a single point with an approximate diameter of 200 μm. This definition of intensity, however, does not consider the transmission of the sound wave through tissues, which, in physical terms, are referred to as media with different acoustic impedances that the wave passes through.

We perceive that the difference in the effectiveness of ESWT power between a superficial tendon such as the ECRB [60] and a deeper tendon such as the insertional supraspinatus is also related to the greater amount of tissue that the sound wave must penetrate in deeper tendons. For these deeper tendons, a high-power EFD may be necessary to prevent the dissipation of energy in tissues with different acoustic impedances.

The variation in tendon thickness may contribute to the differences in efficacy observed among different power levels applied to various tendons. Specifically, the common extensor tendon (CET) measures 3.8 mm in thickness [12], whereas the supraspinatus tendon measures 12 mm [61]. Thicker tendons, such as the supraspinatus, possess greater mass and thickness, resulting in higher density. Consequently, they may require a higher energy level from the shockwave to effectively penetrate and produce a therapeutic effect. Conversely, lower-power shockwaves may lack the necessary intensity to traverse the entire structure of thick tendons and reach problematic areas. In our study, we found that a lower-energy level was needed to penetrate the entire thick tendon structure.

As ultrasonography studies have shown, even a slight angle of incidence change in probe position from perpendicular (≤2°) results in emitted sound wave variation at the surface of interest [62,63,64], possibly also altering the ESWT effects.

We compared the high-energy ESWT and low-energy ESWT for patients with chronic LE. Low-energy ESWT usually has few side effects and good effectiveness, as confirmed by the results of laboratory research [65,66]. Low-energy ESWT may also prevent the phenomenon of lowering the level of adaptation of the patients or the patients’ giving up the treatment because of pain in the middle of the treatment course [67]. Low-energy ESWT is also known to create less local swelling and tenderness [68]. The histological reaction to the ESWT is known to be dose-dependent on the total energy delivered to the tissue [65]. In fact, energy density determines clinical outcomes [59]. It has been shown that shockwaves at low-energy density produce stimulatory effects on cell cultures and enhances wound healing, while shockwaves of high-energy density may inhibit cell growth and interfere with repair potential due to cell destruction and necrosis [69,70]. Therefore, the most effective treatment outcome can only be achieved by using optimal treatment parameters. This is the concept of a ‘window effect’ of treatment effectiveness [23]. In discussing physical energy therapies in the field of musculoskeletal pathologies, it has been shown that the effects of ESWT for knee osteoarthritis [60] and supraspinatus tendinopathy [23] are dose dependent. This has also been noted for low-power laser therapy [71,72,73,74,75]. However, for ESWT in chronic LE, there was no prior evidence of this. Following the results of our study, it is hypothesized that in chronic LE, the ESWT effects are dose dependent, with low power showing better clinical outcomes. However, these results need to be confirmed in randomized controlled trials.

We should start thinking about the physical energies we use in rehabilitation, as ESWT, in the same way that we consider the use of drugs, which possess a minimum effective dose that must be identified through studies.

In chronic LE treated with ESWT alone, the low-energy group (0.10 mJ/mm^2^) showed more effective and had longer-lasting results compared to those treated with a higher energy level (0.20 mJ/mm^2^).

## 5. Limitations

This study had several limitations that should be acknowledged. First, as a retrospective study, it inherently lacks the control and randomization of prospective trials, which may introduce selection bias. Second, not all potential outcomes were assessed, particularly the absence of pre- and post-treatment imaging follow-up, duration of symptoms, type/activity at work, previous conservative treatments, and BMI, which would have provided valuable objective data to correlate with the clinical findings. Third, the assessment scales used in this study, such as pain and functional scores, are subjective and can be influenced by individual patient perception, potentially affecting the reliability of the results. Recent literature has proposed different methods of multimodal sensorimotor evaluation of the hand and forearm, such as combining muscle mechanical properties, pressure pain thresholds, active range of motion, maximal isometric strength, and manual dexterity, which could be suggested for a more holistic view of the sample. This comprehensive approach could guide clinicians in monitoring the recovery of upper extremity injuries more effectively [76]. Future studies should assess the influence of placebo effects on therapy by standardizing treatment protocols including the use of the same language and commands, consistent non-verbal communication, uniform setting and therapist attire, a consistent ritual from welcome to farewell, and blinding participants to the ultrasound image [77]. Future studies with larger sample sizes, randomized controlled designs, and outcomes are needed to validate and expand upon our results.

## 6. Conclusions

Our study demonstrated that low-energy ESMW (0.10 mJ/mm^2^) was more effective than high-energy ESWT (0.20 mJ/mm^2^) for the management of chronic LE treatment, providing greater pain relief and functional improvement over time. These findings support the preferential use of low-energy ESWT in clinical practice for patients with this condition. Future prospective studies and randomized controlled trials with larger sample sizes are warranted to confirm these results and to further explore the underlying mechanisms contributing to the differential efficacy observed between the two treatment modalities. There is a strong relationship between health-related quality-of-life metrics (PRTEE scores) and patient-reported measures (VAS scores), indicating that reductions in pain are closely associated with improvements in functional abilities and overall quality of life.

## Figures and Tables

**Table 1:** Comparison of the outcome measures before treatment in each group. Values are reported as the mean ± standard deviation for continuous variables and as distribution for categoric variables and the *p* value.

Variables	High Energy Group (*n* = 22)	Low Energy Group (*n* = 24)	*p* Value
Age, year	49.3 ± 9.8	50.2 ± 10.4	0.779
Gender, M = 1 F = 2	1 = 13; 2 = 9	1 = 11; 2 = 13	0.369
VAS T0, continuous	6.8 ± 1.1	7.3 ± 1.1	0.112
PRTEE T0 continuous	49.6 ± 2.1	52.2 ± 3.2	0.515

VAS: Visual analog scale; PRTEE: Patient-Rated Tennis Elbow Evaluation.

**Table 2:** Comparison of the outcome measures after treatment in each group. Values are reported as the mean ± standard deviation and the *p* value.

Variables	High Energy Group (*n* = 22)	Low Energy Group (*n* = 24)	*p* Value
VAS T1, continuous	4.4± 0.1	3.6 ± 0.3	0.02
PRTEE T1 continuous	34 ± 6.5	27.1± 11.3	0.01
VAS T2, continuous	3.2± 0.2	2.1 ± 0.2	0.002
PRTEE T2 continuous	24.8 ± 7.4	17.5 ± 8.4	0.003

VAS: Visual analogue scale; PRTEE: Patient-Rated Tennis Elbow Evaluation.

**Table 3:** Comparison of the outcome measures before and after treatment in each group: correlation analysis.

Variable	Correlation with	Coefficient (r)	*p*-Value
VAS T0	PRTEE T0	0.7	<0.001
VAS T1	VAS T2	0.5	<0.05
VAS T1	PRTEE T1	0.7	<0.001
VAS T1	PRTEE T2	0.6	<0.01
VAS T2	PRTEE T1	0.7	<0.01
VAS T2	PRTEE T2	0.8	<0.001
VAS T1	Low-power group	−0.332	0.024
VAS T2	Low-power group	−0.446	<0.002
PRTEE T1	Low-power group	−0.339	0.032
PRTEE T2	Low-power group	−0.510	0.001

## Data Availability

The datasets used and the data analyzed during the current study will be made available upon reasonable request to the corresponding author (G.S.).

## Abbreviations

CET	Common extensor tendon
ECRB	Extensor carpi radialis brevis
EDC	Extensor digitorum communis
EFD	Energy flux density
ESWT	Extracorporeal shockwave therapy
LE	Lateral epicondylitis
NSAIDs	Nonsteroidal anti-inflammatory drugs
PRTEE	Patient-Rated Tennis Elbow Evaluation Questionnaire
US	Ultrasonography
VAS	Visual analog scale
